# Integration of human and mouse single-cell transcriptomes of the developing cerebellum nominates cells-of-origin for group 3 and 4 medulloblastoma

**DOI:** 10.1186/s12885-026-16006-1

**Published:** 2026-04-18

**Authors:** Ian Cheong, Leo Lau, Shraddha Pai

**Affiliations:** 1 Ontario Institute for Cancer Research, Toronto, Canada; 2 Department of Medical Biophysics, University of Toronto, Toronto, Canada; 3 University of Waterloo, Waterloo, Canada

**Keywords:** Bioinformatics, Developing cerebellum, Developmental cellular origins of medulloblastoma, Genomics, Groups 3 and 4 medulloblastoma, Human-mouse integration, Single-cell transcriptomics, Unipolar brush cells

## Abstract

The cerebellar rhombic lip neurogenic niche is critical for glutamatergic neurogenesis, and dysregulated differentiation of rhombic lip lineage cells is hypothesized to cause medulloblastoma (MB), a malignant pediatric cancer lacking targeted therapies. Humans have structural compartmentalization of the rhombic lip into a subventricular zone not seen in mice, and the common Group 4 subtype of MB is hypothesized to arise by dysregulation of EOMES^+^ unipolar brush cells (UBC) generated by this compartment. However, it is unknown whether humans have unique UBC populations not seen in the mouse and, if so, whether Group 4 MB tumour cells resemble these UBC populations. We integrated 336,598 human and mouse single-cell transcriptomes of the developing cerebellum (11–21 post-conception weeks in humans and E10–P14 in mice) and identified two subpopulations of UBC that are enriched in the human samples, relative to mice. One of these populations predominates at 11 post-conception weeks, upregulates *RBFOX1* and *FOXP2*, is predicted to be driven by *OTX2*, *SOX4*, and *SOX11*, and is enriched for axonogenesis and neurodifferentiation pathways. We analyzed 27,735 single-cell transcriptomes from eight MB tumours and recapitulated the observation that Group 4 MB cells best resemble UBC. We then found that two-thirds of Group 3 and Group 4 MB UBC-like cells best resemble human-enriched *RBFOX1*-rich cells. Gene regulatory network analysis revealed that top regulators of Group 4 MB tumour cells include *EOMES*, *SOX4*, and *SOX11*. Our work provides initial evidence for human-enriched UBC states and suggests that *SOX4* and *SOX11* may drive neurogenesis in UBC and gene expression in a subset of Group 4 MB tumour cells. Our findings shed light on genetic determinants of human cerebellar expansion and suggest that human models may be needed to recapitulate the oncogenesis of Group 4 MB.

**Supplementary Information:**

The online version contains supplementary material available at 10.1186/s12885-026-16006-1.

## Introduction

The cerebellum forms part of the hindbrain and is located below the cerebrum and behind the brain stem and fourth ventricle. Like the cerebrum, the cerebellum is divided into two hemispheres which are connected via the vermis, and it contains a cortical layer less than 1 mm thick in adults [1]. The cerebellar cortex itself is further divided into three layers: the molecular layer (outermost), the Purkinje cell layer, and the granule layer (innermost) [2]. Functionally, the cerebellum plays an important role in many day-to-day activities, including motor control, memory, speech and language, visuospatial processing, and executive function [3].

Almost all that is known about cerebellar development comes from studies in mice. Initially, the cerebellar anlage is formed by the isthmic organizer through Fgf8 signalling around embryonic day (E) 8.5 [4, 5]. This is followed by the formation of the cerebellar ventricular zone (VZ) and the cerebellar rhombic lip (RL), the two main progenitor zones which give rise to all neurons of the cerebellum. The cerebellar VZ is specified by expression of *Ptf1a* and gives rise to all GABAergic neurons, including Purkinje cells and Pax2^+^ interneurons [4, 5]. The Purkinje cells arise around E10.5–E13.5 and form the Purkinje cell layer of the cortex, with dendrites that also project into the molecular layer [2, 4]. Meanwhile, the cerebellar RL is specified by expression of *Atoh1* and gives rise to all glutamatergic neurons such as granule cells (GCs) and unipolar brush cells (UBCs) [4, 5]. The cerebellar RL first gives rise to the granule cell progenitors (GCPs) around E12.5, which migrate to form the external granule layer of the cerebellum [4, 6]. Within the external granule layer, the postnatal proliferation and expansion of the GCPs is driven by Shh secreted from the Purkinje cells, before they finally migrate through the Purkinje cell layer and differentiate into GCs to form the (internal) granule layer [4, 5]. RL production of UBCs, marked by expression of *Eomes*, occurs around E13.5. The UBCs first migrate into the developing white matter before finally settling in the granule layer around postnatal day P10 [7].

On the other hand, while the mouse provides a good starting point for understanding cerebellar development in humans, the process in humans is more complex and prolonged. Compared to mice, the surface area of the human cerebellum is 750-fold larger [5]. Furthermore, whereas the adult mouse cerebellum contains approximately 60% of all neurons in the brain, the adult human cerebellum contains approximately 80% of all neurons; this is equivalent to around 70 of the 86 billion neurons in the human brain [5, 8]. Human cerebellar development begins at around 30 days post-conception and continues until around two years after birth [6]. Similar to mice, the human cerebellar VZ and RL give rise to GABAergic and glutamatergic neurons, respectively. Neurogenesis in the cerebellar VZ occurs until around 10 weeks post-conception (PCW), after which it begins to thin, and the cerebellar RL begins to expand. Several weeks later, the cerebellar RL embeds itself into the posterior-most lobule of the cerebellum where it persists until birth [6]. In contrast, the mouse cerebellar RL is much more transient and disappears by E17.5 [6]. Additionally, the human cerebellar RL is elaborated into a rhombic lip ventricular zone (RL-VZ), which is KI67-rich and SOX2^+^, and a rhombic lip subventricular zone (RL-SVZ), which is KI67-rich and SOX2-sparse [6]. The RL-VZ and RL-SVZ are separated by a vascular bed, and progenitor cells in the RL-SVZ generate both the GC and UBC cell lineages [6]. This elaboration of the RL may be a feature unique to human neurodevelopment, as it is not seen in mice or even in rhesus macaques [6].

Given the protracted and unique process of cerebellar development, especially in the cerebellar RL, humans may be particularly susceptible to insults causing various developmental diseases and disorders [5, 9]. Medulloblastoma (MB) is hypothesized to result from failed differentiation during cerebellar development. Neurodevelopmental disorders, such as some forms of autism spectrum disorder, attention deficit-hyperactivity disorder, and developmental dyslexia, are associated with abnormalities in the cerebellum [9, 10]. Furthermore, genes that are implicated in Dandy-Walker malformation—a congenital disorder characterized by hypoplasia of the cerebellar vermis—and autism spectrum disorder show enriched expression across a number of cerebellar cell types [11, 12]. As such, an improved understanding of the human-specific aspects of cerebellar development may provide valuable insights into the origins of these neurodevelopmental disorders.

MB is the most common malignant pediatric cancer of the central nervous system, accounting for approximately 20% of all childhood brain tumours and over 60% of all embryonal tumours [13, 14]. It primarily affects children aged 0–9 at a rate of approximately 5 per million people, although cases do occur in adolescents and adults [13, 14]. Overall, males are approximately 1.7 times more likely to be affected than females [14]. Treatment for MB generally involves with maximal safe resection of the tumour, followed by craniospinal irradiation and adjuvant chemotherapy with a combination of vincristine, cisplatin, cyclophosphamide, and lomustine [15, 16]. Unfortunately, while patient survival may be as high as 80% post-treatment, survivors generally suffer a reduced quality of life and have an increased risk of hearing loss, reduced motor coordination, and cognitive and intellectual deficits [17–19].

Molecularly, MB has been classified into four subgroups (WNT, SHH, Group 3, and Group 4), with each subgroup exhibiting unique gene expression patterns, mutations, DNA methylation profiles, and prognoses [15, 20]. WNT and SHH MB are so named because they are driven by aberrant activation of the WNT and Sonic Hedgehog signalling pathways, respectively [20]. WNT MB makes up only around 10% of cases and has the best prognosis of all subgroups, with 5-year survival reaching up to 95% [21]. Over 85% of WNT MB patients have a mutation in the gene coding for β-catenin, *CTNNB1 *[22]. The SHH subgroup encompasses approximately 30% of all MB cases, and prognosis is generally worse than that of the WNT subgroup [21]. Genes involved in the involved in the SHH pathway are frequently mutated (*PTCH1*, *SUFU*, *SMO*) or amplified (*GLI2*) [22]. SHH tumours have been shown to be transcriptomically most similar to GCP lineage cells, and these cells are hypothesized to be the cell of origin for this subgroup [23].

Meanwhile, despite the fact that the Group 3 and Group 4 MB subgroups account for over 60% of cases, their molecular drivers remain poorly defined [21]. Known driver genes of Group 3 and 4 MB include *MYC*, *MYCN*, *GFI1/B*, *SNCAIP*, *KDM6A*, and *PRDM6*^21,22,24^. Preclinical models are severely lacking for these subgroups, especially for Group 4 MB, which has no cell lines [15]. A better understanding of the dysregulated genes driving Group 3 and Group 4 MB may help with the generation of useful models [15, 16]. Recent studies have found that the transcriptomes of Group 3 and Group 4 MB tumours closely mirror those of the UBC lineage and its progenitors [23, 24]. In particular, these tumours resemble UBCs and, notably, cells of the RL-SVZ [6, 24, 25]. Alterations in the core binding factor alpha (CBFA) complex have been found in Group 3 and Group 4 MB tumours; two of the CBFA complex members, *CBFA2T2* and *CBFA2T3*, are specifically expressed in the RL-SVZ [24]. Additionally, expression of *CBFA2T2* is inhibited by the transcription factor OTX2, and the *OTX2* gene is highly expressed in Group 3 and Group 4 MB [24]. Knockdown of *OTX2* in Group 3 MB cell lines, a proxy for Group 4 MB which lacks preclinical models, resulted in an upregulation of differentiation markers and *CBFA2T2* expression, suggesting that *OTX2* overexpression or loss of function of the CBFA complex could prevent differentiation and drive tumour formation [24, 25]. Differentiation of SHH and Group 3 MB tumour cells can also be induced by thyroid hormone [26], which is required for cerebellar development, further supporting the hypothesis that MB arises from dysregulated differentiation during cerebellar neurogenesis. Although the CBFA complex may be a key player in RL-SVZ differentiation and MB initiation, fewer than 60% of Group 4 MB tumours can be explained by mutations in the complex [24], and more work is needed to identify other developmental programs promoting proliferation of these tumours.

Taken together, this suggests that the gene regulatory networks (GRNs) and molecular drivers of normal cerebellar development, when dysregulated, could be the same drivers of MB tumourigenesis; however, these developmental regulators are poorly understood. Furthermore, the fact that the RL-SVZ is a region of the cerebellum which is uniquely expanded in humans relative to mice leaves open the possibility that UBC cell states not readily apparent in the developing mouse cerebellum, but more abundant in the developing human cerebellum, may be the putative cells-of-origin for Group 3 and 4 MB. Therefore, understanding these GRNs could pave the way for identifying biomarkers unique to these human cell states or developing targeted therapies for tumour cells arising from these populations. We hypothesized that the UBC lineage of the developing human cerebellum contains cell states either missing or less abundant in the mouse, and that the GRNs from human-enriched cell states would resemble those in Group 4 MB cells. We therefore integrated single-cell transcriptomes of developing human and mouse cerebella to identify and characterize UBC cell states enriched in humans, relative to mice. We then analyzed single-cell transcriptomes from MB tumours to ascertain if Group 4 MB tumours resemble human-enriched cell states and what transcription factors these may have in common.

## Methods

### Datasets

This study involves secondary analysis of previously published data that is publicly available. Human samples had already been deidentified in these data. Similarly, this study only involves secondary analysis of mouse data, and no animals were used to generate new data for this work. Therefore, no ethics approval was obtained for this study for either human or animal research.

#### Developing cerebellum

Aldinger et al. performed snRNAseq using nuclei isolated from pulverized frozen cerebellum or from entire dissected cerebellum per a previous protocol [12, 27]. Sepp et al. either sampled entire halves of the cerebellar primordia or representative fragments, such as 25% of the primordium cut perpendicular to its long axis (11 PCW). For mid-gestation samples (17–20 PCW), fragments of the cerebellum were sampled [28].

The counts matrices of two human and two mouse cerebellar single-cell RNA-sequencing (scRNA-seq) datasets [12, 23, 28] were downloaded from the links in the published papers. Low quality cells or nuclei were filtered out as published (Supplementary Fig. 1). Briefly, cells with percent mitochondrial genes that exceeded four standard deviations from the median, were excluded [23]. Similarly, cells with less than 200 genes were excluded, as were cells with genes that exceeded 4 standard deviations from the median. For the human datasets, only cells from samples aged 11–21 PCW were included. We created a single naming system to consolidate cell type labels across all datasets (Supplementary Tables 1–2), a result which yielded a single EOMES^+^ cluster as the starting point for UBC analysis (Fig. 2d).

#### Medulloblastoma

MB scRNA-seq counts from Vladoiu et al. [23] were kindly provided by Dr. Liam Hendrikse and Dr. Jiao Zhang (Dr. Michael Taylor’s lab). The dataset contained 27,735 cells from eight MB tumours: two SHH tumours, two Group 3 MB tumours, and four Group 4 MB tumours. Counts from all cells were combined and normalized using *SCTransform *[29]. Batch correction was performed using fastMNN [30]. To visualize the cells, the top 50 MNN-corrected principal components (PCs) were used for dimensionality reduction with uniform manifold approximation and projection (UMAP).

### Analysis of human developmental hindbrain single-cell transcriptomes

#### Isolation of unipolar brush cells

Following the initial QC described above, we prepared an integrated dataset consisting only of human cells using canonical correlation analysis. Dimensionality reduction (principal component analysis (PCA) and uniform manifold approximation and projection (UMAP)) was performed on the combined cells, and cells were clustered using Louvain clustering. Multiple clustering resolutions were explored and an optimal clustering resolution of 0.8 was selected for being the lowest resolution that identified the *WLS*^+^ *KI67*^+^ rhombic lip cluster. Clusters were then annotated based on expression of known markers and the original cell type annotations from the two datasets (Supplementary Fig. 2a–c, Supplementary Table 1).

#### Unipolar brush cell subclustering

Starting with the subset of rhombic lip lineage cells, we subset for an inferred “unipolar brush cells” cluster (UBC), selecting clusters with the highest expression of *EOMES*. Only cells with non-zero raw counts for *EOMES* were included in the subclustering; 4,587 cells met this criterion. To identify clusters, we used the shared nearest neighbour method and tested multiple clustering resolutions using ranging from 0.1 to 1.0, in increments of 0.05. We used clustree [31] to identify the highest resolution at which clusters were stable. From this, we selected a clustering resolution of 0.5, which resulted in nine clusters. One of these clusters was comprised of cells from a single sample in one of the two datasets used and was excluded from further analysis.

#### Differential expression analysis and pathway enrichment

**Fig. 1:**
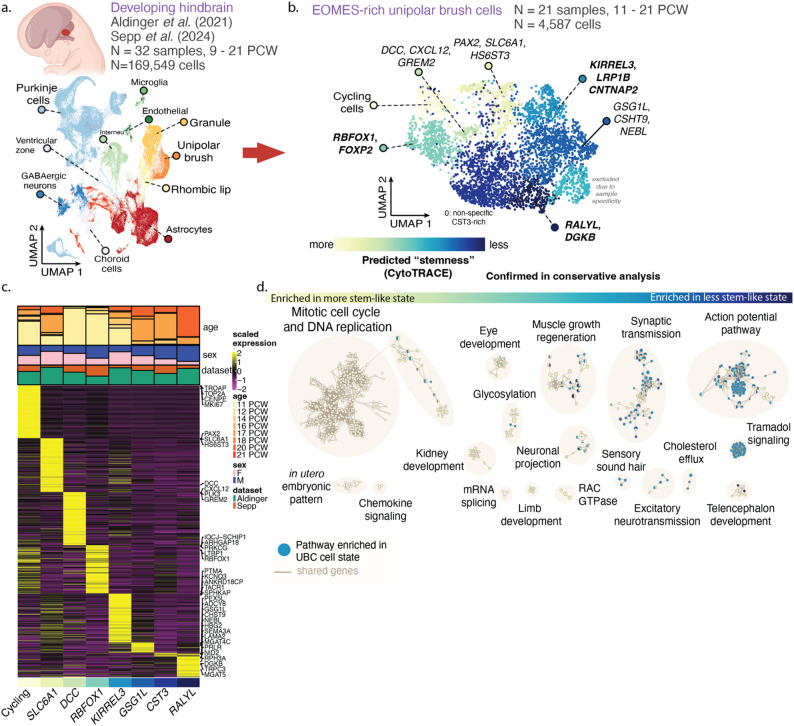
Characterization of human unipolar brush cell clusters from the developing hindbrain. **a** Integrated single-cell transcriptomics from human mid-gestation hindbrain. **b** Reclustered *EOMES*^+^ unipolar brush cells (UBC; 4,587 cells, 20 samples) annotated by marker genes or dominating pathway enrichment theme. Cluster colours indicate cluster median of stemness as measured by CytoTRACE. **c** Top upregulated genes in each fetal UBC cluster. Barplots at the top show cluster-level distribution of gestation age, sex, and source dataset. Heatmaps show cluster-wise Z-scores for marker genes (log-fold change > 2). Clusters in boldface are concordant in a conservative analysis of concordant clusters of *PAX6*^+^*EOMES*^+^ cells across datasets. **d** Pathways enriched in upregulated genes for UBCs. Nodes indicate individual significant pathways, and edges connect pathways with shared genes. Nodes are coloured by the UBC clusters for which the pathways are significant

To identify markers unique to each cluster, one-versus-all differential gene expression was performed for each cluster. For this, we used Seurat [32] *FindMarkers* with the following parameters: *assay = “SCT”*,* logfc.threshold = 0*,* min.pct = 0.01*,* test.use = “MAST”*,* latent.vars = c(“sex”*,* “dataset.name”)*. Genes with an adjusted *p*-value < 0.05 were deemed to be differentially expressed. Markers for each cluster, as shown in the heatmap in Fig. 1c (*ComplexHeatmap) *[33], were defined as differentially genes with a log_2_ fold change greater than 1.5.

The *gprofiler2* R package, which provides access to the online g: Profiler tool [34, 35], was used to run a functional enrichment analysis (*gost*) on upregulated genes (average log_2_FC > 0, nominal *p*-value < 0.05). For the background set, we used all genes that were tested for differential expression analysis for that given cluster. A custom Enrichment Map gene set was used (downloaded from https://download.baderlab.org/EM_Genesets/August_08_2023/Human/symbol/36*)* which included pathways from GO Biological Processes (excluding those inferred from electronic annotation (IEA)) [37], HumanCyc [38], MSigDB (hallmark, C2, and C3 collections) [39, 40], NCI-Nature [41], NetPath [42], Panther [43], Pathbank [44], Reactome [45], and WikiPathways [46]. Only pathways with 10–250 genes were included (𝑛 = 8,404 pathways). The genes that were tested for differential expression were used as the background set for pathway analysis. Pathways with a Benjamini-Hochberg FDR < 0.05 were deemed significantly enriched. Network visualization was performed using Cytoscape [47] v3.10.2 with EnrichmentMap v3.5.0 and AutoAnnotate v1.3.5 [36].

#### Trajectory inference using CytoTRACE and Monocle

To infer relative states of cell differentiation, we used the *CytoTRACE* [48] (Cellular Trajectory Reconstruction Analysis using gene Counts and Expression) v0.3.3 R package to run an integrated *CytoTRACE* (iCytoTRACE) across both datasets. We additionally inferred pseudotime using *Monocle 3* [49, 50]. For this, we ran a PCA (*preprocess_cds*) on the Seurat “integrated” assay and used the top 25 PCs for dimensionality reduction with UMAP (*reduce_dimension* with *umap.min_dist* = 0.3 and *umap.n_neighbor* = 30 L). The trajectory was then learned (*learn_graph*) and cells were ordered along pseudotime (*order_cells*) by setting the root as the cell with the highest CytoTRACE score.

### Joint analysis of human and mouse datasets

#### Dataset integration

To prepare the datasets for integration, the genes in the mouse datasets were unified with the human genes by identifying one-to-one orthologs (*N* = 16,755) using the *biomaRt* R package [51]. Each dataset was individually normalized using *SCTransform *[29] with differences in the cell cycle phase regressed out. This regression controls for artefactual clustering due to cell cycle differences, and is a standard part of removing technical variation that is not biologically meaningful for the context under study [52]. Dimensionality reduction was performed to visualize each dataset separately, as described in the next section.

Canonical correlation analysis (CCA) in Seurat [53] was used to integrate the human and mouse cerebellar datasets (“full cerebellum integration”) (Supplementary Fig. 2). To visualize the integration, dimensionality reduction was performed as described in the next section. Based on the consolidated cell types (Supplementary Table 2), the glutamatergic cells of the rhombic lip (RL) lineage (“RL”, “UBC/GCP progenitor”, “UBC”, “GCP”, and “GC”) and non-neuronal reference cell populations (“endothelial”, “microglia”, and “oligodendrocyte/OPC”) were isolated. Additionally, since expansion of the cerebellar RL occurs after 10 PCW [6], human cells between 7 and 10 PCW were filtered out. Dimensionality reduction was performed on the isolated cells, and the cells were clustered using Louvain clustering (Seurat *FindClusters*, resolution = 0.4; “RL lineage integration” with 26,094 human cells and 55,449 mouse cells). The clusters were then annotated based on cell type marker expression and original cell type annotations (Supplementary Table 2).

The effect of CCA integration was compared to that with simple concatenation of the two datasets, and with integration using two additional approaches, reciprocal principal component analysis (RPCA) from Seurat [53] and Harmony [54] (Supplementary Figs. 3–4). Overall, RPCA appeared to correct for species differences similar to CCA, whereas Harmony fully integrates cells from both species (Supplementary Fig. 2e, Supplementary Fig. 4). However, when considering the originally annotated ventricular zone of the rhombic lip (RL-VZ) and subventricular zone of the rhombic lip (RL-SVZ) cells from the Aldinger dataset [12, 24], the RL-SVZ cells no longer cluster together in Harmony (Supplementary Fig. 4e–f). On the other hand, both CCA and RPCA appear to better preserve the biology of the RL, with the majority of the RL-SVZ cells clustering together. All downstream analyses were based on CCA integration.

#### Dimensionality reduction and clustering

Dimensionality reduction was performed using Seurat. In general, principal component analysis (PCA) was used to determine the top 100 PCs whenever dimensionality reduction was performed (*RunPCA*). The variance explained by each PC was plotted on an elbow plot, and based on the plot, the top 50 (full cerebellum integration) or 25 PCs were used for further dimensionality reduction with UMAP (*RunUMAP*).

To identify subclusters of unipolar brush cells (UBCs), the UBC cluster was isolated, and dimensionality reduction and Louvain clustering were performed. To determine the ideal number of subclusters, the UBCs were clustered at multiple resolutions ranging from 0.1 to 0.8 (Seurat *FindClusters*) and a clustering tree was generated to visualize the flow of cells between clusters across different resolutions (*clustree* package) [31]. Overclustering on the clustering tree appears as the formation of new clusters from multiple “parent” clusters, indicating instability in the clusters [31]. Given that a resolution of 0.4 showed signs of overclustering, a resolution of 0.3 (with six UBC subclusters) was used for downstream analyses (Supplementary Fig. 5).

The human enrichment of UBC subclusters was also replicated using RPCA [55]. For this, human and mouse rhombic lip cells for all datasets were first integrated using RPCA in Seurat. *EOMES*^+^ clusters were then subset from this, and dimensionality reduction (PCA), community detection (*FindNeighbours*), and clustering (*FindClusters*) was run on the “integrated” layer to obtain UBC cluster assignments using RPCA; a cluster resolution of 0.1 was selected as it gave a comparable number of clusters as the CCA.

#### Differential gene expression and pathway enrichment analysis

Starting from the human and mouse RL lineage integration, we subset the human UBCs and performed one-versus-all differential gene expression for each of the UBC subclusters (Seurat). Prior to differential gene expression analysis, genes were first filtered to keep only those that were expressed in at least 1% of all UBCs in each of the human datasets [12, 28]. 8,122 genes remained after filtering. Each dataset was then individually normalized using *SCTransform* with differences in the cell cycle phase being regressed out. The normalized datasets were combined and prepared for differential gene expression analysis (*PrepSCTFindMarkers*). Differentially expressed genes were identified by performing a Wilcoxon rank-sum test comparing cells from one UBC cluster to cells from all other UBC clusters combined; this analysis was performed separately for the Aldinger and Sepp datasets (*FindConservedMarkers*). Genes were only tested if they were expressed in at least 1% of cells in at least one of the tested groups (*min.pct = 0.01*). The resulting nominal *p*-values were corrected using the Bonferroni method, and genes with an adjusted *p*-value < 0.05 were considered significant. Differentially expressed transcription factors (TFs) were identified using the list of 1,639 human TFs from Lambert *et al. *[56].

Differential expression analysis was also performed for each human UBC cluster, using RPCA integration. For this, the human-mouse dataset was subset for human cells, and human cells were re-integrated using *SCTransform*, with differences in dataset being regressed out. Normalized datasets were combined and prepared for gene expression analysis (*PrepSCTFindMarkers*). For this replication analysis, *FindMarkers* was run on the combined set of UBCs from both datasets (*test.use* = “MAST”, *latent.vars* = “dataset”, *logfc.threshold = 0.2*, *min.pct = 0.2*). Genes with an adjusted *p*-value < 0.05 were deemed significant.

Pathway enrichment was performed using *gProfiler2* as described for the human-only analysis. In this instance, only genes upregulated in both human datasets were considered as foreground (log_2_FC > 0 and nominal *p*-value < 0.05 for both the Aldinger and Sepp human datasets). Pathway enrichment analysis of the human genes without a one-to-one mouse ortholog (“non-orthologous genes”) was performed in a similar manner. For this analysis, the non-orthologous genes were set as the query and all human genes from both human datasets, Aldinger [12] and Sepp [28], were used for the background.

#### Inferring developmental stage with pseudotime

Pseudotime analysis was performed on the human RL cells, UBCs, granule cell progenitors (GCPs), and granule cells (GCs) using Monocle 3 [49]. After subsetting the cells from the cross-species integration, PCA was performed to determine the top 100 PCs (*preprocess_cds*). UMAP was run on the top 25 PCs (*reduce_dimension*) with the minimum distance (*umap.min_dist*) set to 0.3 and the size of the local neighbourhood (*umap.n_neighbors*) set to 30. The trajectory of the cells was inferred (*learn_graph*) and the cells were ordered by pseudotime (*order_cells*) by setting the RL cell cluster as the starting point.

### Testing for differences in cell type proportions

To test for differences in cell type proportions, the *scProportionTest *[57], *Milo *[58], and *propeller *[59] packages were used. Briefly, *scProportionTest* performs a permutation test (𝑛 = 10,000 permutations) to determine the 95% confidence interval for the log_2_FD where the FD (fold difference) is the ratio of the cell type proportion in humans to the cell type proportion in mice. Cell types with a |log_2_FD| > 1 and an FDR < 0.05 were considered to be enriched in one species or the other. Meanwhile, *propeller* uses sample-level proportions (biological replicates) to perform a moderated *t*-test using an empirical Bayes framework. As information about sample identity was not available for the Vladoiu dataset, cells were grouped by age, and cells from each age were considered to be a sample. Cell clusters with a Benjamini-Hochberg FDR < 0.05 were considered to be enriched in one species or the other.

*Milo *[58] computes differential abundance between conditions (here, species) in spatial neighbourhoods identified through k-nearest neighbour clustering. It models cell count by biological replicates, thus accounting for sample variability. *Milo* was run on the “integrated” layer for differential abundance testing of UBC spatial neighbourhoods. Spatial neighbourhoods were computed by setting *k = 50*, *d = 30*, and setting conditions to “1_mouse” and “2_human” to force human-enriched neighbourhoods to have a positive sign for log-fold change. Results were similar for other values of *k*. The *nHoods* matrix of the *Milo* object was used to identify identifiers of cells in significant neighbourhoods (cell *i* was a member of neighbourhood *j* if *nHoods[i*,* j] = 1*), so these could be mapped back to the original six UBC clusters. Neighbourhoods with a Spatial FDR < 0.05 were deemed differentially abundant.

### Projecting developmental signatures onto tumour cells with SingleR

The CCA-integrated human and mouse RL lineage cell dataset was used as the developmental reference for projection onto tumour cells after being subset to include only human cells (26,094 cells). Prior to using SingleR [60] for the projection, gene expression in both the tumour and reference datasets were individually normalized using *NormalizeData* on the RNA slot. *SingleR* was then run with *de.method* set to “wilcox”, using the integrated cell type label for projections. Group 3 and Group 4 MB tumour cells that mapped to “UBC” cells (UBC-like tumour cells) were then subset for further projection (11,483 tumour cells). For the projection of the detailed UBC states, the reference dataset was subset to only include the human cells in the six UBC cell clusters uncovered in this work (UBC_i, i = {0, … 5}) (6,747 cells). This reduced human UBC-specific reference set was then projected onto the UBC-like tumour cells to ascertain similarity to the new UBC clusters.

### Gene regulatory network inference with pySCENIC

Gene regulatory network inference was performed using *pySCENIC *[61]. Briefly, raw counts were used to infer co-expression modules of TFs and corresponding target genes using *GRNBoost2*. *cisTarget* was then used to predict regulons by searching for enriched TF-binding site motifs 10 kbp upstream and downstream of the transcription start site of the target genes. The activity of each regulon in each cell was then measured with *AUCell*, and the regulon specificity score (RSS) was calculated to identify the top regulons that are specific to each cluster/cell type.

## Results

### Integration of human-only cerebellum scRNAseq reveals eight unipolar brush cell populations in the developing cerebellum

We integrated published human cerebellum single-cell transcriptomes [12, 28] (> 169,000 cells, 32 samples), and isolated cells of the rhombic lip lineage (29,668 cells) (Fig. 1a). From these we reclustered 4,587 *EOMES*^+^ cells, which we deemed as unipolar brush cells (UBC; 20 samples, 11–21 PCW; Figure. 1b). Reclustering revealed eight populations of UBC that lie along an axis of progressive neuronal differentiation [48]. We identified marker genes distinguishing these clusters and which pathways upregulated genes were enriched in (Fig. 1c–d, Supplementary Tables 3–4). Consistent with neurodifferentiation processes, the cells with the highest stemness signature showed an upregulation of genes involved in cell cycle processes (*TOP2A*, *MKI67*, *CENPE*), suggesting that these are cycling progenitor cells. Pathway enrichment analysis revealed a gradual shift in enriched pathways over the course of predicted neurodifferentiation, from the earliest cell clusters demonstrating enrichment of cell proliferation pathways, to the later cell clusters showing enrichment of processes of mature neuronal function, such as synaptic transmission (Fig. 1d).

As a complementary approach, we performed a conservative UBC clustering separately on each input dataset and with added stringency filters requiring non-zero expression of *PAX6* in each cell, in addition to non-zero expression of *EOMES* (240 cells in total in Aldinger, 244 in Sepp). *PAX6* is an established marker of the rhombic lip lineage [5, 12, 62, 63], and the goal was to exclude any *EOMES*^+^ cells potentially from deep cerebellar nuclei. In this scenario, we identify three clusters in each dataset, and identify a reciprocal best match for each cluster across three datasets (Supplementary Fig. 6, Supplementary Tables 5–7). This analysis identified a *KIRREL3*-enriched cluster (Jaccard similarity = 0.32), an *RBFOX1*-enriched cluster (Jaccard similarity = 0.19), and a less defined cluster enriched for *RALYL* and *DGKB* (Jaccard similarity = 0.11) (Supplementary Fig. 6i, Supplementary Table 7). From this analysis, we conclude that there are at least four UBC populations in the 11–21 PCW age period: an *MKI67*-rich cycling population; an *RBFOX1* and *FOXP2*-rich early population; a population upregulating *KIRREL3*, *LRP1B*, and *CNTNAP2*; and a later population upregulating *RALYL* and *DGKB*.

To identify mouse UBC populations, we performed a similar clustering analysis of *Pax6*^+^*Eomes*^+^ cells using the two mouse datasets in this study. (*n* = 313 cells in the Sepp dataset; 3,345 cells in the Vladoiu dataset; Supplementary Fig. 7, Supplementary Tables 8–10). We found a weak best-match (Jaccard similarity = 0.06) of a cluster upregulating *Cxcl12*, *Robo2*, and *Syt1*. Other matches were weaker still (Jaccard similarity < 0.05). *Rbfox1* and *Foxp2* are not in the set of concordantly upregulated genes (Supplementary Table 10).

### Integration of human and mouse developing cerebellar single-cell transcriptomes reveals human enrichment of unipolar brush cells

**Fig. 2:**
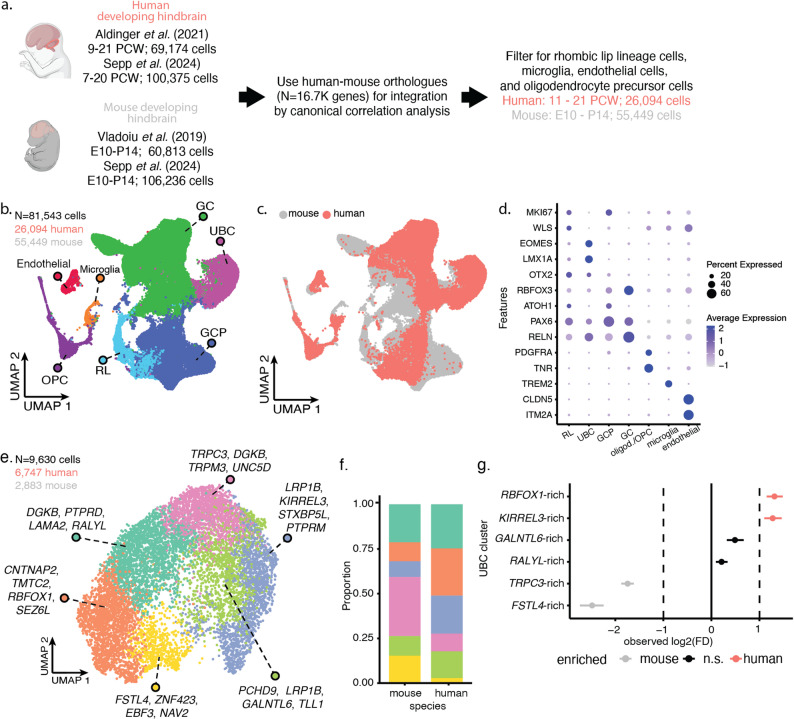
Integration of human and mouse UBCs reveal two UBC populations disproportionately present in humans. **a** Schematic for human-mouse transcriptomic integration. **b–c** UMAP visualization of integrated human-mouse cerebellar RL lineage cells and non-neuronal reference cells coloured by cell type (**b**) and species (**c**). **d** Expression of marker genes for RL lineage cells and non-neuronal reference cells in the integrated data. **e** UMAP visualization of subclustered UBCs. **f** Proportion of human and mouse cells in each of the UBC clusters. **g** Relative differences in proportion for each UBC cluster between humans and mice. Clusters with a log_2_FD > 1 are significantly enriched in humans, and vice versa for cell types with log_2_FD < − 1 (FDR < 0.05, *n* = 10,000 permutations) [57]

We then integrated the two human cerebellar datasets with two mouse cerebellar datasets using canonical correlation analysis (CCA) (*n* = 336,598 cells in total; 169,549 human, 167,049 mouse; Fig. 2a, Supplementary Table 1, Supplementary Fig. 1a–d) [12, 23, 28, 53]. Overall, the cells clustered together by cell type and not by dataset or species (Supplementary Fig. 2e–g). In comparison, the unintegrated datasets showed clear dataset and species batch effects (Supplementary Fig. 3). As we were primarily interested in the glutamatergic neurons that arise from the rhombic lip (RL), we isolated the RL lineage cells and three non-neuronal reference populations, and performed dimensionality reduction for visualization. As before, the human and mouse cells appeared to be well-integrated (Fig. 2b–c, Supplementary Fig. 8). We identified 18 clusters, with clusters generally containing cells from multiple datasets (Supplementary Fig. 8d). The clusters were then annotated using known cell type markers (Fig. 2b, d), such as *EOMES* and *LMX1A* for unipolar brush cells (UBC), *WLS* for RL cells, and *RBFOX3* for granule cells (GC) (Fig. 2b, d, Supplementary Fig. 9). The annotation of the clusters was verified using the original cell type annotation. We included non-neuronal cell populations of endothelial cells, microglia, and oligodendrocytes/oligodendrocyte precursor cells in the integration, to determine potential under-integration of human and mouse cells. Given that these non-neuronal cells from both species clustered together, it appears that CCA is appropriately correcting for the species batch effect (Fig. 2b-c).

Comparing RL-lineage cell type proportions between mice and humans revealed an enrichment of UBCs in humans (Supplementary Fig. 10). Across all the datasets, the UBCs make up 29% of the human RL-lineage cells compared to only 5.5% in mice. The enrichment of UBCs in humans was found to be significant by two different approaches (Q < 0.001, Supplementary Fig. 10b-c) [57, 59]. Meanwhile, the proportion of RL cells and granule cells (GCs) were not significantly different between the two species (log_2_FD < 1, Supplementary Fig. 10b). Granule cell progenitors (GCP) appeared to be enriched in mice by some measures (Supplementary Figs. 10b-c). This effect is likely seen because the data analyzed includes early postnatal timepoints in mice when GCP expansion occurs, but does not include human cells from the third trimester onwards, when a similar expansion occurs in the developing human cerebellum [5].

### Enrichment of unipolar brush cell subpopulations

**Fig. 3:**
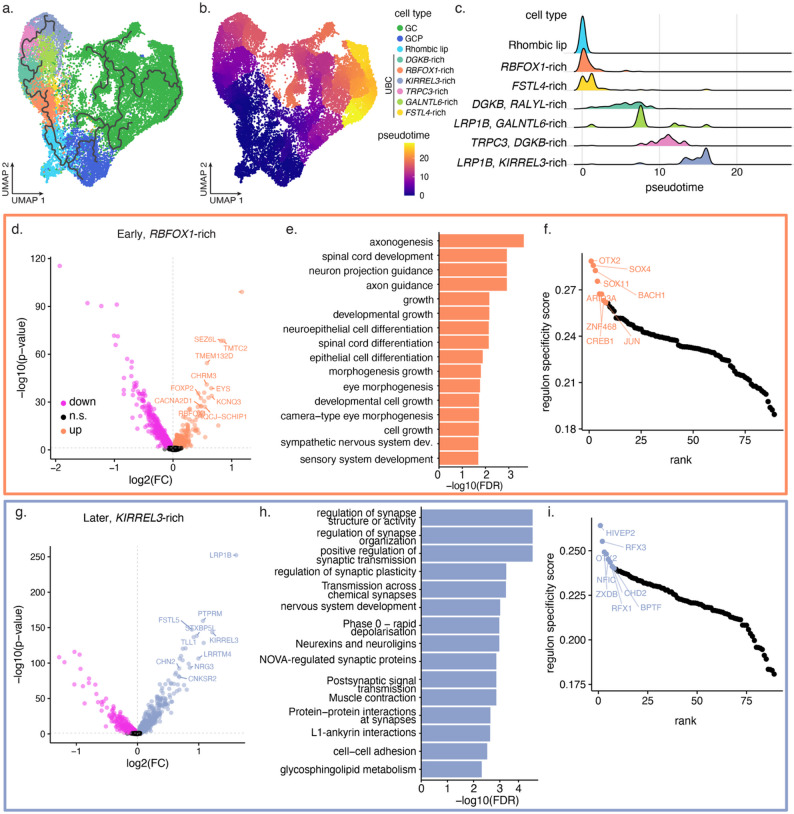
Characterization of human-enriched UBC cell states. **a–b** UMAP visualization of the human cerebellar RL lineage cells coloured by cell type (**a**) and inferred pseudotime (**b**) (*n* = 23, 048 cells). The RL cells were set as the root cells and the inferred trajectory is overlaid in (**a**). **c** Distribution of inferred pseudotime for cells of the unipolar brush cell lineage. **d** Differentially expressed genes in the human-enriched *RBFOX1*-rich cluster; top upregulated genes are labelled. Data shown for the Aldinger dataset. **e** Top enriched pathways in genes upregulated in the *RBFOX1*-rich cluster (Q < 0.05). **f** Top regulons for the *RBFOX1*-rich UBC cell cluster. **g** Differentially expressed genes in the *KIRREL3*-rich cluster; top upregulated genes are labelled. Data shown for the Aldinger dataset. **h–i** Enriched pathways (**h**) and top regulons (**i**) for KIRREL3-rich UBC cell cluster

Given that the UBCs were enriched in humans compared to mice, we wondered if certain subclusters of UBCs were driving this enrichment. We subclustered UBCs and identified six UBC subpopulations (Fig. 2e). To annotate these, we performed differential expression analysis of the human cells in these clusters, identifying markers conserved in both datasets (Figs. 2 and 3, Supplementary Table 11). These clusters recapitulated markers identified from the human-only analysis. We identified an *RBFOX1-*rich population, two loosely defined *LRP1B*-rich populations, one of them enriched in *KIRREL3*, a *DGKB* and *RALYL*-rich population, and others. We performed species-specific differential abundance testing for each UBC cluster and found that the *RBFOX1*-rich and the *KIRREL3*-rich clusters are enriched in humans compared to mice (Fig. 2f-g; Q < 0.001, log_2_FD > 1). In particular, the *RBFOX1*-rich cells comprise 26.4% of human UBCs compared to 10.6% of mouse UBCs (Fig. 2f). Similarly, the *KIRREL3*-rich cells comprise 21.3% of human UBCs compared to 8.8% of mouse UBCs (Q < 0.001 and log_2_FD > 1). This human enrichment of UBC clusters was also replicated using a method that tests for differential abundance at higher granularity of cellular neighbourhoods and accounts for cell counts per replicate [58]. By this approach, about half of the cell neighbourhoods (282 of 559) show increased abundance in humans relative to mice, including > 99% of cells belonging to the *RBFOX1*-rich and *KIRREL3*-rich cell clusters (Spatial FDR < 0.05 and log-fold change > 0; Supplementary Fig. 11, Supplementary Table 12). Using a third method that relies on per-sample proportions [59], the *RBFOX1*-rich UBC cluster was not found to be significantly enriched, but the *KIRREL3*-rich cell cluster was (*RBFOX1*-rich cluster: Q > 0.1; *KIRREL3*-rich cluster: Q < 0.025; Supplementary Fig. 13). To ensure that the enrichment of UBCs was not due to the choice of integration method, we also integrated human and mouse cells using RPCA [55]. UBC clustering similarly identifies an *RBFOX1*-rich cluster, a *LRP1B-* and *KIRREL3*-rich cluster, and a *RALYL-* and *DGKB*-rich cluster (Supplementary Fig. 12, Supplementary Tables 13–14). By this approach, we also identify a broad enrichment of human UBCs, including in the *RBFOX1*-rich and *KIRREL3*-rich clusters (Supplementary Fig. 12b). We conclude from these analyses that multiple types of UBCs are enriched in humans, notably the *RBFOX1*-rich and *KIRREL3*-rich clusters.

In humans, the majority of UBCs at 11 PCW appears to be of the *RBFOX1*-rich cluster, whereas the proportion of *KIRREL3*-rich cells increases and forms the majority of the human cells at 20 PCW, along with the *RALYL*- and *DGKB*-rich clusters (Supplementary Fig. 14a). On the other hand, the earlier UBCs in mice are comprised of *RALYL*-rich cluster and an *FSTL4*-rich cluster, whereas the *KIRREL3*-rich and *DGKB*-rich clusters cell populations arise peri- and postnatally (Supplementary Fig. 14a). The UBC subpopulations do not appear to be driven by any single dataset; rather, they seem to be well-represented in both datasets (Supplementary Fig. 14c). Similarly, the distribution of UBC subpopulations by sex appears to reflect the sex distribution of cells by dataset (Supplementary Fig. 15) and does not appear to be driven by any one sex. Considering only UBC cells from the Aldinger dataset, which are more evenly balanced in terms of sex as compared to the Sepp dataset (Aldinger: 2,915 cells, 58.5% female; Sepp: 3,832 cells, 5.2% female), 60.0% of cells of the *RBFOX1*-rich subpopulation is from female samples and 69.2% of the *KIRREL3*-rich subpopulation is from female samples (Supplementary Fig. 15).

### Characterization of human-enriched unipolar brush cells

To better understand the temporal distribution of the *RBFOX1*-rich cells, we inferred the developmental trajectory and pseudo-temporal ordering of human cells in the RL lineage [49]. Consistent with previous analyses [4, 12, 23], the RL trajectory splits into two to give rise to both GCs and UBCs (Fig. 3a). The *RBFOX1*-rich cells appear to arise directly after the RL cells, and the *KIRREL3*-rich cells appear at the terminal end of the trajectory, consistent with the changes in proportion seen between 11 and 20 PCW (Fig. 3b–c, Supplementary Fig. 14).

A total of 73 genes were upregulated in the *RBFOX1*-rich population of both Aldinger and Sepp datasets (7,928 genes tested; log_2_FC > 0 and Q < 0.05; Fig. 3d, Supplementary Fig. 16, Supplementary Table 11). This list included neurodevelopmental genes such as *CNTNAP2* and *FOXP2*, mutations in both of which have been linked to various intellectual and speech disorders [64, 65]. Pathway enrichment analysis showed that genes upregulated in this cell cluster are enriched for pathways related to neuronal and axonal development (34 enriched pathways; FDR < 0.05, g: Profiler [66]; Fig. 3e, Supplementary Table 15). The *OTX2* gene regulatory network was found to be the most active in the *RBFOX1*-rich cells (pySCENIC; Fig. 3f). Other top regulons included those of *SOX4*, *SOX11*, *BACH1*, and *CREB1* (Fig. 3f, Supplementary Table 16). *OTX2* has been shown to be overexpressed in MB and is hypothesized to maintain a less differentiated state in the RL [21–24].

Cells in the *KIRREL3*-rich UBC cluster showed an upregulation of 459 genes, including *NRG3*, *CNKSR2*, and *KIRREL3 *[67–69], genes involved in neuronal differentiation and mutations in which are associated with neurodevelopmental disorders (Q < 0.05, log_2_FC > 0; Supplementary Table 11). Consistent with the fact that these cells arise later in development, genes upregulated in *KIRREL3*-rich cells were enriched for pathways related to synaptic activity (Fig. 3h; Supplementary Table 17). Top regulons enriched in the *KIRREL3*-rich UBC cluster included *OTX2*, but also *RFX3*, a transcription factor that serves as a master regulator of nervous system development and ciliogenesis, and disruption in which causes some types of autism [70–72] (Fig. 3i, Supplementary Table 16). Taken together, these results suggest that the *RBFOX1*-rich cells represent a cell state that is relatively less differentiated than the *KIRREL3*-rich cells.

### Shared drivers in unipolar brush cells and medulloblastoma

**Fig. 4:**
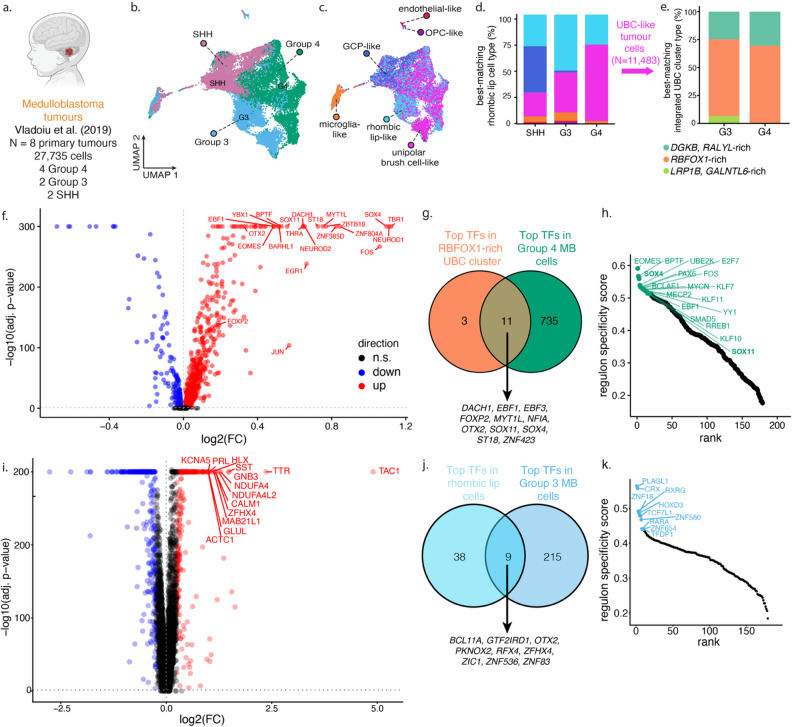
Group 3 and 4 MB tumour cells resemble human-enriched RBFOX1-rich UBC. **a** Single-cell transcriptomic dataset of MB tumours [23]. **b–c** UMAP visualization of MB tumour cells, coloured by tumour subtype [23] (**b**) and best-matching RL cell type (**c**). Reference dataset includes non-neuronal cells. **d** Proportion of MB tumour cells stratified by subtype and best-matching developmental cell type. **e** Proportion of UBC-like Group 3 and 4 MB tumour cells stratified by subtype and best-matching UBC subtype. UBC subtypes are as those shown in Fig. 2**e**, **f** Differentially expressed genes in Group 4 MB compared to SHH and Group 3 MB; the top upregulated genes are labelled. Dotted lines indicate a log_2_ fold change (FC) of 0 and an adjusted *p*-value of 0.05. Genes with an adjusted *p*-value of 0 are shown at the top of the plot with their adjusted *p*-value set to 1 × 10^− 300^. **g** Number of transcription factors that are upregulated in Group 4 MB tumour cells and in *RBFOX1*-rich UBC cells. **h** Top regulons driving Group 4 MB cell state. **i **Differentially expressed genes in Group 3 MB compared to SHH and Group 4 MB. **j** Number of transcription factors upregulated in Group 3 MB tumour cells and in rhombic lip cells. **k** Top regulons in Group 3 MB tumour cells. GCP, granule cell progenitor; OPC, oligodendrocyte precursor cell; n.s., not significant

To ascertain if the human-enriched UBC states above are similar to Group 3 and 4 MB tumour cells, we analyzed published single-cell transcriptomes of 27,735 cells from SHH, Group 3, and Group 4 MB tumours (Fig. 4a–b) [23]. We first determined the developmental best-matching cell state for each tumour cell, using human cells from our integrated dataset of cerebellar RL lineage cells and non-neuronal reference cells (Fig. 4c–d). Nearly half of SHH tumour cells best resemble granule cell progenitors (42.4%); in contrast, < 2% of Group 3 MB (1.8%) or Group 4 MB cells do (1.1%). By comparison, over half of Group 3 MB tumour cells best resemble the RL progenitor cells (51.1%), and roughly one-third resemble UBCs (36.6%). Two-thirds of Group 4 MB tumour cells resemble UBC cells (69.8%), while most of the remaining cells resemble RL cells (27.4%) (Fig. 4d). This profile of best-matching developmental cell states is consistent with the current model of developmental MB oncogenesis, which hypothesizes that SHH arise from dysregulated differentiation of GCPs, while Group 3 and 4 MB tumours arise from dysregulation of the RL, with Group 3 MB arising from a less differentiated cell state than Group 4 MB [23–25, 73]. The similarity of a fraction of SHH tumours to the unipolar brush cell lineage has been documented in previous studies [23, 24]. To further identify which of the six integrated UBC cell states Group 3 and 4 MB tumour cells best resemble, we projected signatures of the subclustered UBC populations onto UBC-like Group 3 and 4 tumour cells (11,483 tumour cells, 6,747 reference UBC cells). We found that two-thirds of Group 3 MB (68.6%) and Group 4 MB (69%) tumour cells best resemble the *RBFOX1*-rich UBC cell state, with the *DGKB*/*RALYL*-rich cell state accounting for roughly one-third of cells in each tumour type (24.7% Group 3 MB, 30.5% Group 4 MB cells; Fig. 4e); this distribution does not appear attributable to any individual tumour sample (Supplementary Fig. 17). While a small fraction of Group 3 and Group 4 MB cells mapped to the *LRP1B/GALNTL6*-rich cell state (6.7% Group 3 MB, < 1% Group 4 MB), none of the cells mapped to the later, *KIRREL3*-rich UBC cell state (Fig. 4e). In summary, Group 3 and 4 MB cells that are UBC-like appear to best resemble the early *RBFOX1* cell state.

To determine if Group 4 MB tumour cells share drivers of gene expression—*i.e.*, transcription factors (TFs)—with the *RBFOX1*-rich cell state, we next examined TF expression in these tumour cells (Supplementary Table 18). A total of 746 upregulated TFs were identified in Group 4 MB tumour cells (log_2_FC > 0 and Bonferroni-corrected Q < 0.05; Fig. 4f). Of these, 11 TFs are also upregulated in the *RBFOX1*-rich UBC cells, including regulators of neurogenesis such as *SOX4*, *SOX11*, *OTX2*, and *FOXP2* (Fig. 4g). We next inferred top regulons in Group 4 MB tumour cells (Fig. 4h, Supplementary Table 19). The UBC marker *EOMES* was one of the top active regulons for the Group 4 MB cells, consistent with previous research demonstrating the similarity of Group 4 MB tumour cells to UBCs in the developing cerebellum [24]. Similar to the *RBFOX1*-rich UBC, *SOX4* and *SOX11* were also among the top regulons for Group 4 MB tumour cells (Fig. 4h).

As Group 3 MB cells best match rhombic lip cells, we performed a similar analysis of top regulons in these tumour cells and the comparison to TFs also upregulated in the rhombic lip (Fig. 4i–k). The top regulons in Group 3 MB tumour cells include those of PLAGL1, CRX, and TFDP1. A total of 9 TFs were upregulated in both, Group 3 MB tumour cells and rhombic lip cells. These include *OTX2*, *ZIC1*, *RFX4*, and *BCL11A*.

## Discussion

In this work, we identified a population of *RBFOX1*-rich UBCs that is enriched in humans, arises early in development relative to the parent cerebellar RL progenitor cells, and expresses genes related to development and neurogenesis. This population of cells may be driven by the gene regulatory action of SOX4 and SOX11 and transcription factors such as FOXP2. Overlapping top regulons, shared upregulated transcription factors, and the single-cell projection data collectively suggest the possibility that the *RBFOX1*-rich UBC cluster is a cell-of-origin for some subtypes of Group 4 MB tumours.

As part of this work, we performed some clustering of human unipolar brush cells in mid-gestation (11–21 PCW), which revealed cell states in different states of proliferation or maturation. One of the clusters we identify is a cycling *EOMES*^+^ population, which likely includes progenitor cells from the rhombic lip subventricular zone, a region known to produce TBR2^+^ MKI67^+^ cells (TBR2 is the protein form of *EOMES*) [24]. Because *EOMES* is also expressed by deep cerebellar nuclei, we performed a conservative analysis of UBC clustering, limited to cells with non-zero expression of *PAX6*, a rhombic lip lineage marker. This filter resulted in the identification of three broad clusters, concordant across the two datasets tested (Supplementary Fig. 6). These clusters appear to have markers indicating varying degrees of neurodifferentiation. For instance, *RBFOX1* and *FOXP2*, upregulated by an early UBC cluster, are known regulators of human neurodevelopment, although to our knowledge, these have not previously been noted as marker genes in the context of rhombic lip lineage cells [74–76]. Similarly the later UBC cluster upregulates genes such as *NLGN1*, *ANK3*, and *KIRREL3*, which are involved in the structural organization and function of neuronal synapses in excitatory neurotransmission and are also linked to genetic risk of cognitive or neuropsychiatric disorders [67, 77–79]. A third UBC cluster upregulates *DGKB* and *RALYL*; *DGKB* promotes neuronal maturation by regulating dendritic growth and spine maturation [80, 81]. In all, the UBC clusters identified appear to show different stages of neuronal maturation. However, the window of gestation examined here, 11 to 21 PCW, may be too narrow to capture all stages of human UBC differentiation. An expanded study of UBCs across the human lifespan coupled with immunohistochemical validation of marker genes will likely be necessary to identify distinct UBC cell types.

While to our knowledge role of *SOX4* and *SOX11* in human cerebellar development has not been studied, both genes have been shown to be important in the expansion and differentiation of progenitor cells in the mouse cerebral cortex [82]. For example, knockout of *Sox11* in mice results in reduced sizes of the cerebellum and the cerebral cortex [83]. Interestingly, *Sox4* activates *Eomes *[82], and a similar mechanism of UBC lineage commitment may be present in the developing human cerebellum. Future work could test the impact of *SOX4* overexpression or downregulation in UBC cell states. Separately, the spatial distribution of the UBC cell states identified here, including the states that are statistically enriched in humans, remains to be resolved. Single molecule fluorescence in situ hybridization (smFISH) in primary tissue from the developing human hindbrain could be performed to verify expression of subcluster-specific markers; for example, *RBFOX1*, *FOXP2*, *SOX4,* and *KIRREL3*.

An alternative explanation for the presence of the human-enriched UBCs could be temporal and spatial differences in sampling procedures for the source human and mouse datasets used in this work, particularly the human dataset. Given the much shorter timescale of development in mice compared to humans, there may be transient cell states in mouse cerebellar development that may not be present in the mouse datasets due to the lack of sampled timepoints. Integration of gene signatures from the *RBFOX1*-rich or *KIRREL3*-rich cells with spatial assays (e.g., FISH, spatial transcriptomics) of the developing human cerebellum may identify distinct spatial distributions of the UBC clusters, in turn suggesting different models of UBC lineage specification, and disambiguating between *EOMES*^+^ UBCs and deep cerebellar nuclei [84]. Separately, UBCs are not uniquely found in the cerebellum, as a population of UBCs also exists in the dorsal cochlear nucleus of the brainstem [7]. As the nuclei for the human datasets used in our study were extracted from cerebellar fragments, contamination from other regions of the hindbrain is unlikely.

A technical consideration arises from the choice of harmonization algorithm for the human-mouse datasets. While the integration shown here was performed using canonical correlation analysis (CCA) in Seurat [53], integration was also performed using reciprocal principal component analysis (RPCA) [32] and Harmony [54]. In general, integration with RPCA did not appear to give different results from CCA, and similar cell type clusters were identified (Supplementary Figs. 4, 12). However, integration with Harmony appeared to mix cells too strongly, with related but distinct cell types clustering together (Supplementary Fig. 4e–f). Indeed, benchmarking of various methods for integrating cells across different species has shown that Seurat CCA and RPCA are among the top performers in terms of biological conservation and species mixing [85].

A different methodological consideration stems from our use of orthologous genes. Here, we integrated human and mouse cells using genes with one-to-one orthologs. However, evolution has given rise to duplicated and novel genes in humans which may contribute to human-specific features of neurodevelopment [86, 87]. The non-orthologous genes excluded from our integration were significantly enriched for 345 pathways that were related to cell division processes, immune system processes, drug metabolism, and chemical perception and response (Q < 0.05; Supplementary Fig. 18). Given that this study focuses on neurodevelopment, future analyses could benefit from exploring the role of the non-orthologous human genes, particularly those controlling cell proliferation. While some orthologs have been shown to have human-specific expression compared to other mammals [75, 88] and human-specific cell states have been identified using orthologs [28], human genes with no known mouse orthologs may also play a factor in driving the unique expansion of the human cerebellum [86]. As such, whether any non-orthologous genes play a role in promoting expansion and proliferation of the human cerebellum will need to be investigated further.

Using published MB single-cell transcriptomes, we found that *SOX4* and *SOX11* were upregulated in cell clusters of Group 4 MB tumours (Fig. 4f, h). This finding is consistent with previous research showing that these genes were overexpressed in primary MB tumours [89], although this work was published prior to the discovery of the four molecular subgroups of MB, and tumour subtypes were not indicated [20]. Similar to that in the *RBFOX1*-rich cell cluster, *SOX4* and *SOX11* were found to be among the top regulons of Group 4 MB cells (Fig. 4f). Given the role of *SOX4* and *SOX11* in neural proliferation and their high expression in Group 4 MB, we hypothesize that downregulation of these TFs in MB long-passage cell lines will reduce tumour proliferation and promote a more differentiated phenotype. An alternative approach would be to ascertain if overexpression of *SOX4* and *SOX11* in a suitable 2D or 3D human cerebellar neural model [90] prolongs neuronal maturation. Additional mechanistic studies could be performed to better understand the genes, pathways, and downstream effects *SOX4* and *SOX11* in the context of UBC differentiation and Group 4 MB. Altogether, these results may help identify potential targets for therapy as well as potential diagnostic and prognostic biomarkers. We do not exclude the possibility that other TFs shared between the *RBFOX1*-rich UBC cell state and Group 4 MB tumours (Fig. 4g) may be worthwhile of consideration as candidates to develop laboratory models of Group 4 MB oncogenesis. We also note that the size of the dataset used here (six Group 3 and 4 MB tumours) likely does not capture the heterogeneity of these tumours, and this analysis will need to be repeated in a larger tumour dataset and integrated with genetic and epigenetic tumour data to assess generalizability and develop models of developmental oncogenesis.

## Conclusions

In summary, our analysis revealed two subpopulations of UBCs that are enriched in the developing cerebellum of humans compared to mice. Group 4 MB tumour cells appear to share multiple features with one of these, an early *RBFOX1*-rich UBC state. One direction for future work is the molecular characterization and delineation of the UBC cell lineage in humans and mice that would refine the findings here using complementary approaches such as in situ hybridization or immunohistochemistry in primary tissue. Another approach is to use the findings here to validate which TFs have a joint effect on the developing cerebellum as well as support proliferation of MB tumours, using genetic perturbation in preclinical MB models, or 2D or 3D models of the developing cerebellum [90]. Such TFs may be useful in developing novel preclinical models for Group 4 MB to promote rational therapy design.

## Supplementary Information

Supplementary Material 1.

Supplementary Material 2.

## Data Availability

The integrated human UBC and integrated human and mouse UBC Seurat objects have been made available at https://doi.org/10.5281/zenodo.17488151. Software to reproduce the analysis in this manuscript is available at [https://github.com/RealPaiLab/HumanMouse_CBintegration] (https:/github.com/RealPaiLab/HumanMouse_CBintegration) .

## Abbreviations

GCP: Granule cell progenitors
PCW: Post-conception weeks
RL-SVZ: Rhombic lip Subventricular Zone
RL-VZ: Rhombic lip Ventricular Zone
UBC: Unipolar brush cells
